# A Meta-Analysis of Microbial Therapy Against Metabolic Syndrome: Evidence From Randomized Controlled Trials

**DOI:** 10.3389/fnut.2021.775216

**Published:** 2021-12-15

**Authors:** Binhui Pan, Xiujie Liu, Jiangmin Shi, Yaoxuan Chen, Zhihua Xu, Dibang Shi, Gaoyi Ruan, Fangyan Wang, Yingpeng Huang, Changlong Xu

**Affiliations:** ^1^Department of Gastroenterology, The Second Affiliated Hospital and Yuying Children's Hospital of Wenzhou Medical University, Wenzhou, China; ^2^Institute of Ischemia/Reperfusion Injury, Wenzhou Medical University, Wenzhou, China; ^3^Department of Pathophysiology, School of Basic Medicine Science, Wenzhou Medical University, Wenzhou, China; ^4^Department of Gastrointestinal Oncology, The Second Affiliated Hospital and Yuying Children's Hospital of Wenzhou Medical University, Wenzhou, China

**Keywords:** prebiotics, probiotics, synbiotics, microbial metabolites, metabolic syndrome, fecal microbiota transplantation

## Abstract

**Background and aims:** Metabolic syndrome (MetS), accompanied with significant intestinal dysbiosis, causes a great public health burden to human society. Here, we carried out a meta-analysis to qualify randomized controlled trials (RCTs) and to systematically evaluate the effect of microbial therapy on MetS.

**Methods and results:** Forty-two RCTs were eligible for this meta-analysis after searching the PubMed, Cochrane, and Embase databases. Pooled estimates demonstrated that treatment with microbial therapy significantly reduced the waist circumference (WC) (SMD = −0.26, 95% CI −0.49, −0.03), fasting blood glucose (FBG) (SMD = −0.35, 95% CI −0.52, −0.18), total cholesterol (TC) (SMD = −0.36, 95% CI −0.55, −0.17), low-density lipoprotein cholesterol (LDL-C) (SMD = −0.42, 95% CI −0.61, −0.22), and triacylglycerol (TG)(SMD = −0.38, 95% CI −0.55, −0.20), but increased the high-density lipoprotein cholesterol (HDL-C) (SMD = 0.28, 95% CI.03, 0.52). Sensitivity analysis indicated that after eliminating one study utilizing *Bifidobacteriumlactis*, results became statistically significant in diastolic blood pressure (DBP) (SMD = −0.24, 95% CI −0.41, −0.07) and in Homeostatic Model Assessment of Insulin Resistance (HOMA-IR) (SMD = −0.28, 95% CI −0.54, −0.03), while the body mass index (BMI) showed significant difference after eliminating one study utilizing oat bran (SMD = −0.16, 95% CI −0.31, −0.01). There was still no significant effect in systolic blood pressure (SBP) and in hemoglobin A1c (HbA1c%).

**Conclusion:** In patients with MetS, the conditioning with microbial therapy notably improves FBG, TC, TG, HDL-C, LDL-C, WC, BMI (except for the study using oat bran), HOMA-IR, and DBP (except for the Study using *Bifidobacteriumlactis*), however, with no effect in SBP and in HbA1c%.

## Introduction

Metabolic syndrome (MetS) is prevalent in the whole world and holds the largest burden of non-communicable diseases worldwide. It is a metabolic intertwined condition composed mainly of morbidities such as glucose intolerance, dyslipidemia, abdominal obesity, and high blood pressure (1). The development of this metabolic perturbation could double the risk of developing type 2 diabetes mellitus, obesity, cardiovascular disease, non-alcoholic steatohepatitis, and cancer (2, 3). According to the National Health and Nutrition Examination Survey in the U.S., the weighted MetS prevalence has steadily increased from 32.5% in 2011–2012, 34.6% in 2013–2014, and to 36.9% in 2015–2016, respectively (4). Therefore, it is urgent to prevent and control the development of MetS.

Notably, the sedentary lifestyles and the preference for nutrient-depleted, energy-dense, and highly refined foods have been considered as the main etiological factors. However, the corresponding prevention measures did not obtain anticipative results in practices. As the microbiota become the center of systematic diseases, published studies in the last decades have shown that the underlying mechanisms of MetS might have originated from flora disturbance. According to different fiber types (5, 6), fat composition (7, 8), food additives (9, 10), and microbiome could establish different sensitivity, and the individuals with MetS had a lower gut microbiota diversity than the healthy ones (11). *Proteobacteria* and *Firmicutes* (other than *Ruminococcaceae*) were reported to be positively associated with MetS, whereas the Bacteroidetes and *Ruminococcaceae* have a negative association (12).

Therefore, nowadays, microbial therapy that includes microbial agents and fecal microbiota transplantation (FMT), which could modulate intertwined microbiota, has emerged gradually as the new candidate to MetS treatment due to the recently published observations in both animal and human studies of its beneficial effects. In animal experiments, it has been demonstrated that oligofructosein, Lactobacillus fermentum TS1 and S2, pasteurized A. muciniphila, and a combination of Lactobacillus and Bacillus subtilis have shown tremendous potential, especially in lipid metabolism in treating MetS (13–16). In addition, microbial metabolites, such as short-chain fatty acids (SCFAs) contributing to improved glucose homeostasis and insulin sensitivity, were also identified as a therapeutic target for MetS (17, 18).

In the last few decades, FMTs ranging from the healthy to the target-therapy subjects, with the aim of correcting microbiota perturbation, have shown promising metabolic improvements. To begin with, FMT was broadly researched in *Clostridioides difficile* infection (19, 20). Considering that altered gut microbiome may be one of the factors contributing to inflammatory bowel disease (IBD), FMT later became of increasing importance in IBD remission (21–23). More recently, emerging evidence has indicated that MetS is another potential target for FMT therapy. One of the randomized controlled trials enrolled 68 bariatric patients with MetS who were randomly allocated to FMT or placebo group (24). Improvements were seen in Homeostatic Model Assessment of Insulin Resistance (HOMA-IR), insulin sensitivity, and diastolic blood pressure (DBP). Another pilot FMT trial reported that patients in the FMT arm had a decrease in both glucose and insulin level compared to baseline, suggesting a protective role of FMT in MetS (25).

These data suggested that microbial therapy could exert a remarkable benefit to a host with MetS risk factors. However, due to the variety in microbial therapy type and dosage, the interplay between microbial therapy and MetS has not yet been systematically expounded. We decoupled the risk factors for analyses and investigated whether there was a microbial therapy link to hyperglycemia, dyslipidemia, hypertension, and anthropometric parameters; thus, systematically addressing the compelling published studies regarding the effect of microbial therapy on specific risk factors.

## Methods

### Search Strategy

The Preferred Reporting Items for Systematic Reviews and Meta-Analysis (PRISMA) guidelines were followed in this meta-analysis (26). A search of the electronic literature up to May 2021 was conducted using the Pubmed database, the Cochrane Library, and the Embase database. The search strategy was developed with the following keywords and synonyms for related terms: intervention (“prebiotics” OR “probiotics” OR “synbiotics” OR “short-chain fatty acids” OR “niacin” OR “bile acids” OR “bacterial metabolites” OR “fecal microbiota transplantation) AND disease (“metabolic syndrome”). The RCTs examining the effect of microbial therapy on MetS were eligible for this analysis. There was no language restriction. The initial search after importing the located results from the database into the EndNote was derived from the titles and abstracts evaluation in accordance to the appropriateness of our selection criteria. Sequentially, full texts examination was conducted for a better choice to our study question. Two reviewers independently carried it out and then recorded the concrete inclusion or exclusion felts. Any disagreement was resolved by conversation. The bibliographies of all identified related papers were carefully checked to perform a recursive search. We also contacted authors of studies that have incomplete information in available databases to complete the partial texts, which will then maximize our chances to get eligible research.

This measure was also applied for fully published studies that randomized MetS patients to receive microbial therapy or placebo, but did not refer to data concerning subsequent available intervention results, so as to get the data at the most recent point of follow-up.

### Selection Criteria

Randomized controlled trials (RCTs) conducted in MetS human subjects with the intervention of microbial therapy were considered as our inclusion criteria. The MetS diagnosis must meet at least three of the following five criteria in accordance with the International Diabetes Federation Guidelines: (1) Increased waist circumference (WC) with ethnic-specific WC cut-points (White and all other ethnic groups—men ≥ 94 cm; women ≥ 80 cm. South Asians, Chinese, and Japanese—men ≥ 90 cm; women ≥ 80 cm); (2) Triglyceride (TG) ≥ 150 mg/dl (1.7 mmol/L) or treatment for elevated triglycerides; (3) High-density lipoprotein cholesterol (HDL-C) <40 mg/dl (1.03 mmol/l) in men or <50 mg/dl (1.29 mmol/L) in women, or treatment for low HDL; (4) Systolic blood pressure (SBP) ≥ 130, diastolic blood pressure (DBP) ≥ 85, or treatment for hypertension; and (5) Fasting blood-glucose (FBG) ≥ 100 mg/dl (5.6 mmol/L) or after 2 h glucose loading blood glucose was ≥7.8 mmol/L or was previously diagnosed with type 2 diabetes. Availability in data for quantitative calculation was the final eligible criteria. Animal experiments, *in vitro* studies, reviews and meta-analysis, letters, and comments were excluded for this analysis.

### Outcome Assessment

The overriding outcome assessment was the effect of the microbial therapy on MetS included BMI(kg/m^2^), body weight (kg), WC (cm), hip circumference (cm), waist-to-hip ratio, body fat mass (BFM), body fat percentage (BFP) (%), blood pressure (BP) including SBP and DBP (mmHg), FBG (mmol/L), insulin resistance (HOMA-IR) or S (%), TC (mmol/L), HDL-C (mmol/L), low density lipoprotein cholesterol (LDL-C) (mmol/L), TG(mmol/L), and/or HbA1c%. These were some of the commonly used indicators related to our topic.

### Data Extraction

All relevant data from each article were independently examined and extracted by the two authors as dichotomous outcomes to estimate reliability, and some of the concrete information needed were as follows: (1) characteristics of the studies (i.e., the first author, publication year, and number of included participants), (2)' characteristics of the participants (i.e., age, sex, and BMI), (3) information on interventions (i.e., route of administration, dosage, duration of treatment, length of follow-up, and set of control group), and (4) outcome variables (i.e., anthropometric parameters, lipid profile, and glucose metabolism). Disagreements were resolved by consensus and no divergence required adjudication.

### Quality Assessment

One author critically appraised all eligible studies to determine the risk of bias, while a second author critically appraised a random sample of included studies to check for consistency. Conflicts in the quality assessment were resolved by a mutual discussion *via* reference to the original paper. The methodological quality of RCTs was independently assessed by two reviewers using the Cochrane Risk Assessment Scale mentioned in the Cochrane handbook where six items, including selection bias (random sequence generation and allocation concealment), performance bias (blinding of participants and personnel), detection bias (blinding of outcome assessment), attrition bias (incomplete outcome data), reporting bias (selective reporting), and other biases, were evaluated. Reviewers appraising each criterion demonstrated if the included study has conformed to each bias minimization item by recording “high risk,” “low risk,” or “unclear.”

### Statistical Analysis

RevMan 5.3 was used for calculation. SMD with 95% CIs at end-point data from intervention and control groups were measured for continuous variables through DerSimonian and Laird random effects meta-analysis, therefore reflecting the efficacy of microbial therapy treatment. The heterogeneity between the study-specific estimates was qualitatively assessed with Cochran's *Q* test and further quantified by the I^2^ statistics, while the former demonstrated the inconsistency among results and the latter indicated the proportion attributed to the heterogeneity rather than sampling error of total variation in the study estimates. In this, value of *p* < 0.10 or a value of >50% was considered suggestive of significant heterogeneity. When noted heterogeneity existed, possible explanations were investigated *via* subgroup analyses according to some variables, such as the type of microbial therapy. Sensitivity analysis was also conducted by removing one study in turn to estimate the weight of each study in heterogeneity. These measures may partly explain the observed variability so the final conclusion should be made with caution. Publication bias was conducted using Begg's and Egger's tests. If there were ≥10 eligible studies in our eventual analysis, funnel plots would be employed for evidence of asymmetry and, hence, would be a possible publication bias. Review Manager Version 5.3 was used for generating these analyses. A value *p* ≤ 0.05, except for heterogeneity, was considered to have statistical significance.

## Results

### Identification of Eligible Studies

**Figure 1:**
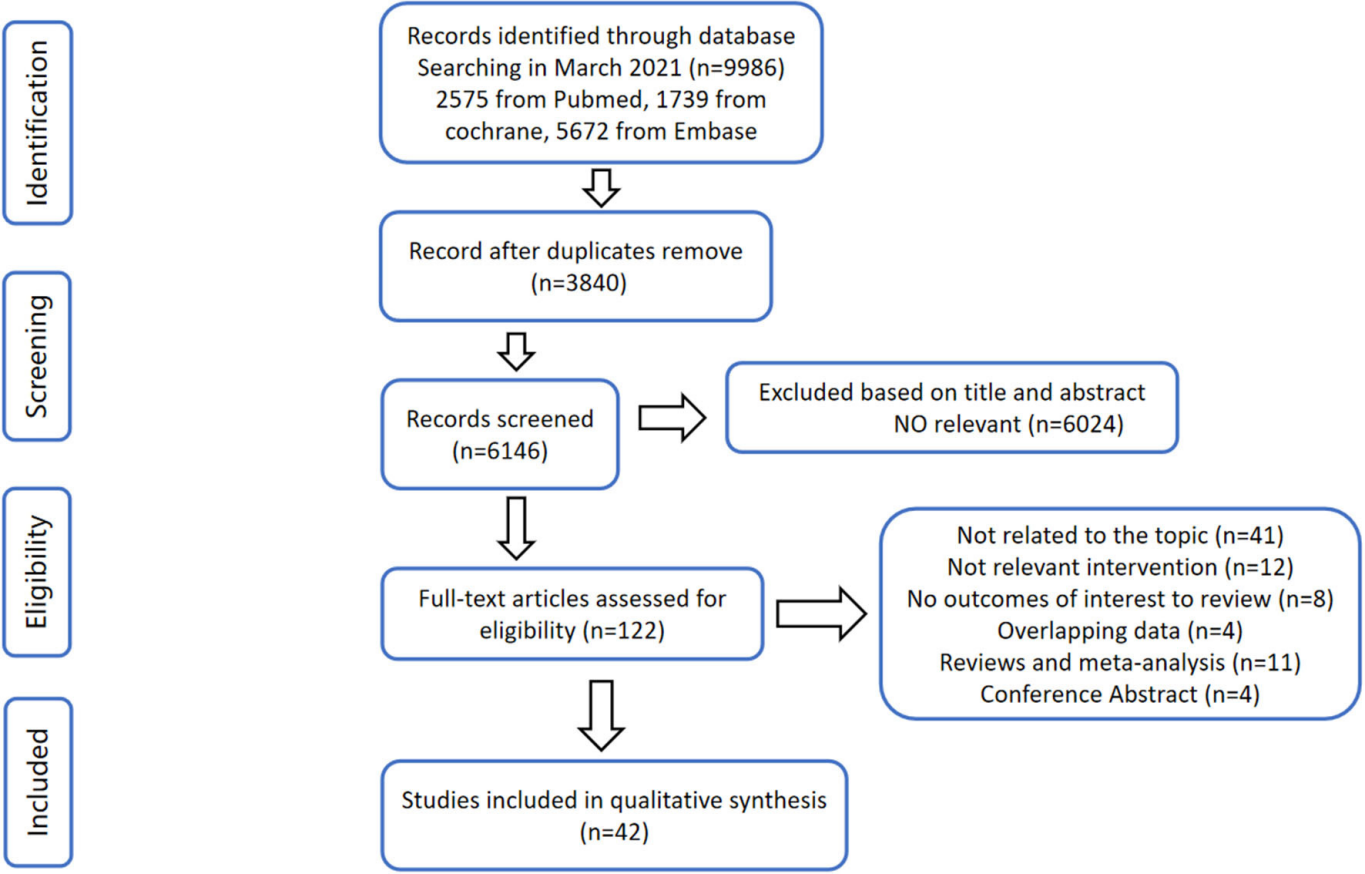
Flow diagram according to the preferred reporting items for systematic reviews and meta-analyses (PRISMA) protocol.

A flow diagram outlining the overall search strategy and selection procedure in this meta-analysis is shown in Figure 1. Among the 9,986 records identified in our initial search, there were 3,840 duplications removed. After being screened for titles and abstracts, 6,024 studies were excluded since they delivered inconformity of information to our subject. For the remaining 122 papers correlated to the topic, 80 studies were ineligible due to its dissociation to the topic (*n* = 41), irrelevant intervention (*n* = 12), null outcome of interest to review (*n* = 8), overlapping data (*n* = 4), reviews and meta-analysis (*n* = 11), and conference abstract (*n* = 4). Finally, the search strategy has returned 42 studies for qualitative synthesis in this meta-analysis.

### Characteristics of Included Studies

The characteristics of included studies are shown in Table 1. Among 42 eligible studies, 14 studies intervened with prebiotics (24, 29–39, 46, 47), 10 with probiotics (40, 41, 43, 48–54), 6 with synbiotics (24, 44, 55–58), 10 with microbial metabolites (27, 28, 59–66), and the remaining 4 with fecal microbiota transplantation (FMT) (24, 25, 45, 67). Among these studies, Mocanu et al. (24) not only explored the respective but also the synergetic efficiency of FMT and the prebiotics on MetS. The earliest paper was published in 2007, while the latest was in 2021. Most studies included were carried out in the Western countries, except for 10 studies that were mainly focused on Asian populations (two in China, two in Korea, one in Palestine, five in Iran) (29, 31, 37, 40, 44, 53, 55, 56, 58, 66). Exceptionally, other researchers such as Bernini et al. (52) utilized *Lactobacillus* as probiotic intervention and chose *Bifidobacteriumlactis, while* Leila et al. (53) used *Lactobacillus* and *Bifidobacterium* for observation (52). For microbial metabolites, one study used whey protein (27), another study utilized *Lactobacillus plantarum* fermented barley (66), which is abundant of biologically active ingredients, and other studies employed niacin as bacterial metabolites. Prebiotics were implicated in researches, such as isomaltulose (46), glucose polysaccharide (37), and resistant starch (24, 32). For FMT intervention, the participants were randomized in receiving the intervention from single lean vegan-donors.

**Table 1:** Characteristics of included studies.

**References**	**Country**	**Participants** **Number (F/M) age**		**Intervention of experimental group**	**Duration**	**Comparison**	**Outcome**
Depommier et al. (15)	Germany	50 (28/22)	35.1 (21–45)	Extended-release niacin; 1,000 mg a day	52 weeks	Placebo	hsCRP↓, LDL-C↓, TG↓, cIMT↓, HDL-C↑, FMD↑, FPG(-), glycosylated hemoglobin(-)
Gouni-Berthold et al. (27)	Germany	180 (85/95)	52.9 ± 10.3;53.9 ± 9.5	Whey protein; 150 g(7 g MPM) twice a day	3 months	Placebo	TG↓, LDL-C↓, FPG↓, HDL-C↑, ApoB(-), TC(-), INS(-), HbA1c(-), WC(-), SBP(-), DBP(-), hsCRP(-)
Gregory (2012)	America	60 (24/36)	46 (40–69)	Extended-release niacin; 2 g a day	16 weeks	Placebo	TG↓, LDL-C↓, VLDL-C↓, TC↓, HDL-C↑
Martin (2018)	France	19 (0/19)	47 ± 13	Extended-release niacin; 2 g a day	8 weeks	Placebo	TG↓, LDL-C↓, ApoB↓, TC↓, hsCRP↓, IL-7↓, VEGF↓, EGF↓, FPG↑, HDL-C↑, INS↑, ApoAI(-), IL-6(-), IL-1α(-), TNF-α(-)
Linke et al. (28)	Germany	60 (18/42)	45.2 ± 3.9	Extended-release niacin; 1,000 mg a day	6 months	No intervention	hsCRP↓, HDL-C↑, TG(-), LDL-C(-), TC(-), FPG(-), WC(-), HbA1c(-), HOMA-IR(-)
Harold (2010)	America	1613 (506/1107)	57.9/57.7/58.7/56.5/57.3/57.5	Extended-release niacin; 1,000 mg a day(T1); 2,000 mg a day(T2)	4 weeks(T1) 20 weeks(T2)	Placebo	HDL-C(-), TG(-), LDL-C(-), SBP(-), DBP(-)
Aaron (2019)	America	35 (24/11)	59.7 ± 10.9 52.3 ± 5.6	Acipimox; 250 mg every 6 h	7 days	Placebo	FFA↓, HDL-C(-), TC(-), TG(-), hsCRP(-), TNFR2(-), MPO(-), HOMA-IR(-), baseline brachial artery diameter(-), flow-mediated dilation(-), nitroglycerin-mediated dilation(-)
Eric (2008)	America	15 (0/15)	46 ± 8(32, 57)	Extended-release niacin; 2 g a day	6 weeks	High-fat meal	TG↓, INS↑
Sony (2017)	America	2067	(18, 45)	Extended-release niacin; 1,500–2,000 g a day	12 months	Statin+placebo	Lp(a)↓, HDL-C(-), TG(-), LDL-C(-), TC(-), HbA1c(-)
Abutair (29)	Palestine	36 (18/18)	47.05 (3.6); 47.50 (4.2)	Psyllium; 10.5 g a day	8 weeks	No intervention	TG↓, LDL-C↓, WC↓, TC↓, FPG↓, SBP↓, DBP↓, HDL-C(-)
Dall'Alba et al. (30)	Brazil	44 (27/17)	62 ± 9	Partially hydrolysed guar gum;10 g a day	6 weeks	No intervention	WC↓, HbA1c↓, UAE↓, TG(-), TC(-), FPG(-), SBP(-), DBP(-), LDL-C(-), HDL-C(-), SBP(-), DBP(-), hsCRP(-), GFR(-)
Daniel (2011)	Germany	20(0/20)	50.7 ± 9.8 (32, 64)	Palatinose (isomaltulose); 50 g	Once	Conventional carbohydrate (glucose syrup/sucrose)	FPG↓, INS↓, TG(-), TC(-), FFA(-), LDL-C(-), HDL-C(-), VLDL-C(-)
Jarrar et al. (31)	The United Arab Emirates	80	28.3 ± 11.8; 25.6 ± 9.9	Gum Arabic; 20 g a day	12 weeks	Placebo (pectin)	HDL-C↑, FPG↓, WC(-), TC(-), LDL-C(-), SBP(-), DBP(-)
Johnston et al. (32)	The United Kingdom	20 (8/12)	(21, 70)	Fiber supplement (resistant starch); 40 g a day	12 weeks	Placebo	Insulin sensitivity↑, HOMA(-)
Kassi (33)	Greece	38 (24/14)	47.3 ± 10.3	Stevia rebaudiana; 4 times a week	4 months	Sweet snack	SBP↓, ox-LDL↓, DBP(-), WC(-), FPG(-), TC(-), HbA1c(-)
Katcher (34)	America	50 (25/25)	(20–65)	Whole-grain; 4–7 servings a day	12 weeks	Refined-grain	CRP↓, WC↓, LDL-C↓, TC↓, HDL-C↓, INS↓, SBP(-), DBP(-), FPG(-), IL-6(-), TNF-α(-)
Lankinen et al., (35)	Finland	106 (54/52)	59 ± 7	Whole-grain; 8–8.5 g/100 g of dietary fiber+16–18 g/100 g of fat a day	12 weeks	Refined-grain	INS(-), FPG(-), HOMA-IR(-), TC(-), HbA1c(-)
Leão et al., (36)	Brazil	154 (113/41)	47.6 ± 12.6	Oat bran (3 g β-glucan); 40 g a day	6 weeks	Low-calorie diet	WC↓, TG↓, HDL-C↓, FPG↓, SBP↓, DBP↓
Lefranc (37)	China	120 (0/120)	(20–35)	NUTRIOSE(a glucose polysaccharide); 34 g a day	12 weeks	Standard maltodextrin	WC↓
Louise (2019)	Denmark	27	(18, 60)	Wheat bran extract (10.4 g/d AXOS); 30 g fiber intake a day	4 weeks	self	WC(-), TG(-), TC(-), FPG(-), SBP(-), DBP(-), LDL-C(-), VLDL-C(-), HDL-C(-), SBP(-), DBP(-), HOMO-IR(-), ApoB(-), INS(-), hsCRP(-)
Mocanu et al., (24)	Canada	68 (60/8)	49 ± 10	Fermentable fiber (resistant starch type IV, soluble corn fiber, acacia gum); 27 g(F)/33 g(M) a day + Fecal microbial transplantation	6 weeks	Non-fermentable fiber	LDL↓, Insulin sensitivity↓, HOMO-IR↑, DBP↑
Robertson et al. (38)	The United Kingdom	15 (7/8)	48.9 ± 3.9	High-amylose maize (HAM-RS2); 40 g a day	8 weeks	Placebo	HOMO-IR↓, FPG↓, INS↓, SBP(-), TG(-), FPG(-), TC(-)
Schioldan et al. (39)	Denmark	19 (5/14)	Not mentioned	Healthy carbohydrate diet; 64 g high dietary fiber+16 g arabinoxylanper+21 g resistant starch+statin a day	4 weeks	Refined carbohydrates+ statin	TC↓, LDL-C↓, HDL-C(-), FPG(-), FFA(-), INS(-), HOMA-IR(-), hsCRP(-), IL-6(-), SBP(-), DBP(-), apoB-48(-)
Carmen (2019)	Spain	53	Not mentioned	Probiotic capsules containing L. reuteri V3401; once a day	12 weeks	Maltodextrin	IL-6↓, sVCAM-1↓, HDL-C(-), FPG(-), INS(-), TC(-), TG(-), LDL-C(-), SBP(-), DBP(-)
Chang et al. (40)	Korea	101 (31/70)	36.45 ± 9.92; 37.16 ± 8.89	A functional yogurt NY-YP901; twice a day	8 weeks	Placebo yogurt	LDL-C↓, WC(-), INS(-), TC(-), TG(-), HDL-C(-), INS(-), SBP(-), DBP(-), HbA1c(-)
Fabiola (2014)	Brazil	24 (24/0)	NFM: 63y (60.5–75.7y)FM: 62y (58.3–67y)	Fermented milk containing L. plantarum; 80 mL a day	90 days	Non-fermented milk	TC↓, FPG↓, IL-6↓, HDL-C(-), WC(-), INS(-), HOMA-IR(-), TC(-), TG(-), LDL-C(-), SBP(-), DBP(-)
Khaider (2013)	Russia	40 (27/13)	52.0 ± 10.9; 51.7 ± 12.1	Cheese containing the probiotic Lactobacillus plantarum TENSIA; 50 g a day	3 weeks	Control cheese	SBP↓, DBP↓, TC(-), TG(-), HDL-C(-), FPG(-), AST(-), ALT(-), Waist-to-hip ratio(-)
Leber et al. (41)	Austria	28 (10/18)	51.5 ± 11.4; 54.5 ± 8.9	Bottles containing L. casei Shirota; 65 ml a day	3 months	No intervention	hsCRP↑, LBP↑, TC(-), TG(-), SBP(-), DBP(-), ALT(-)
Leila (2018)	Iran	44 (22/22)	44.05 ± 6.6; 44.55 ± 5.7	Probiotic yogurt containing Lactobacillus acidophilus La5 and Bifidobacterium lactis Bb12; 300 g a day	2 months	Regular yogurt	VCAM-1↓, FPG↓, INS(-), HOMA-IR(-)
Luciana (2016)	Brazil	51	(18, 60)	Milk containing the probiotic Bifidobacterium lactis HN019; 80 ml a day	45 days	No intervention	TC↓, LDL-C↓, IL-6↓, TNF-α↓, WC(-), INS(-), TG(-), HDL-C(-), INS(-), SBP(-), DBP(-), FPG(-), HOMA(-)
Pan et al. (42)	China	31	(30, 65)	Fermented barley—wheat flour compound noodles; 200 g a day	10 weeks	Whole wheat noodles	TG↓, INS↓, HOMA-IR↓, FPG(-), LDL-C(-), HbA1c(-), WC(-), HDL-C(-), SBP(-), DBP(-), TC(-)
Rikke (2012)	Denmark	50 (28/22)	12.9 ± 1.0; 13.4 ± 1.1	Capsules containing the freeze-dried probiotic strains L salivarius Ls-33 ATCC SD5208	12 weeks	Placebo	FPG(-), HOMA-IR(-), INS(-), WC(-), LDL-C(-), HDL-C(-), SBP(-), DBP(-), TC(-), TG(-), FFA(-), CRP(-), IL-6(-), TNF-α(-)
Tripolt et al. (43)	Austria	28 (10/18)	51 ± 11; 55 ± 9	YAKULT light containing L. casei Shirota; 195 ml a day	12 weeks	Standard medical therapy	sVCAM-1↓, FPG(-), HOMA-IR(-), INS(-), IL-6(-), IL-10(-), TNF-α(-), hsCRP(-), ox-LDL(-)
Vanessa (2015)	Austria	28 (10/18)	51 ± 11; 55 ± 9	YAKULT light containing L. casei Shirota; 195 ml a day	12 weeks	Standard medical therapy	TG(-), TC(-), SBP(-), DBP(-), LDL-C(-), HDL-C(-)
Arrigo (2020)	Italy	60 (33/27)	72 ± 3; 71 ± 3	Bottles containing Lactobacillus plantarum PBS067, Lactobacillus acidophilus PBS066 and Lactobacillus reuteri PBS072 with active prebiotics; one bottle a day	60 days	Placebo	TG↓, TC↓, FPG↓, WC↓, hsCRP↓, TNF-α↓,LDL-C↓, HDL-C↑, HOMA-IR(-), SBP(-), DBP(-)
Karim (2020)	Iran	60 (25/35)	42.33 ± 1.49; 40.6 ± 1.13	Synbiotic capsules containing Lactobacillus casei, Lactobacillus acidophilus, Lactobacillus rhamnosus, Lactobacillus bulgaricus, Bifidobacterium breve, Bifidobacterium longum and Streptococcus thermophiles; one a day	8 weeks	Placebo (containing the same materials plus starch and no bacteria)	TG↓, FPG↓, WC(-), TC(-), SBP(-), DBP(-), LDL-C(-), HDL-C(-), FPG(-)
Safavi et al. (44)	Iran	70	(6, 18)	Synbiotic capsules containing Lactobacillus Casei, Lactobacillus Rhamnosus, Streptococcus Thermophilus, Bifidobacterium Breve, Lactobacillus Acidophilus, Bifidobacterium Longum and Lactobacillus Bulgaricus; one a day	8 weeks	Placebo	WC↓, Waist-to-hip ratio↓, TG↓, TC↓, LDL-C↓, SBP(-), DBP(-), FPG(-)
Samira (2018)	Iran	46 (33/13)	57.1 ± 1.5; 60.8 ± 1.6	Synbiotic capsule containing Lactobacillus casei, Lactobacillus rhamnosus, Streptococcus thermophilus, Bifidobacterium breve, Lactobacillus acidophilus, Bifidobacterium longum, Lactobacillus bulgaricus; two a day	3 months	Placebo capsule contained maltodextrin	FBG↓, INS↓, HOMA-IR↓, PYY↑, TC(-), TG(-), SBP(-), DBP(-), LDL-C(-), HDL-C(-), IL-6(-), hsCRP(-)
Tannaz (2014)	Iran	38 (23/15)	46.79 ± 9.5	Synbiotic capsules containing Lactobacillus casei, Lactobacillus rhamnosus, Streptococcus thermophilus, Bifidobacterium breve, Lactobacillus acidophilus, Bifidobacterium longum and Lactobacillus bulgaricus; two a day	28 weeks	Placebo capsule (250 mg maltodextrin)	FBG↓, HOMA-IR↓, TG↓, TC↓, HDL-C↑, TG(-), LDL-C(-)
Allegretti et al. (25)	America	22 (20/2)	44.5 ± 14.4; 43.3 ± 12.8	Fecal microbial transplantation from a single healthy lean donor	12 weeks	Placebo	FBG↓, HOMA-IR↓
Loek (2018)	The Netherlands	20 (0/20)	55.0 ± 8.2	Fecal microbial transplantation from a single lean vegan-donor	2 weeks	Autologous fecal microbial transplantation	TC(-), TG(-), LDL-C(-), HDL-C(-), FBG(-), INS(-), HbA1c(-), ALT(-), AST(-), CRP(-)
Vrieze (45)	The Netherlands	18 (0/18)	47 ± 4; 53 ± 3	Fecal microbial transplantation from healthy lean donors	6 weeks	Autologous fecal microbial transplantation	Insulin sensitivity↑, FBG(-), TC(-), TG(-), LDL-C(-), HDL-C(-), SBP(-), DBP(-), FFA(-)

*hsCRP, High sensitivity C-reactive protein; LDL-C, low-density lipoprotein cholesterol; TG, triglycerides; cIMT, carotid intima media thickness; HDL-C, high-density lipoprotein cholesterol; FMD, flow-mediated vasodilation; FPG, fasting plasma glucose; MPM, malleable protein matrix; VEGF, vascular endothelial growth factor; Apo, apolipoprotein; TNF-α, tumor necrosis factor alpha; TC, total cholesterol; INS, insulin; HbA1c, hemoglobin A1c; WC, waist circumference; SBP, systolic blood pressure; DBP, diastolic blood pressure; SAE, serious adverse event; VLDL-C, very low-density lipoprotein cholesterol; IL, interleukin; FFA, free fatty acid; TNFR2, tumor necrosis factor receptor 2; MPO, myeloperixodase; HOMA-IR, Homeostatic Model Assessment of Insulin Resistance; Lp(a), lipoprotein (a); UAE, urinary albumin excretion; GFR, glomerular filtration rate; CRP, C-reactive protein; AXOS, arabi-noxylan oligosaccharides; sVCAM-1, soluble vascular cell adhesion molecule 1; AST, aspartate aminotransferase; ALT, alanine aminotransferase; LBP, lipopolysaccharide-binding protein; PYY, peptide YY*.

### Quality of Included Studies

As shown in Table 2, the allocation concealment, blinding of participants and personnel, and incomplete data outcome were the main fields that are reaching a high risk of bias. Nevertheless, most studies were at low risk of bias and of high methodological quality. In 42 trials that reported the effect of microbial therapy on MetS, 20 were judged as fully marked by authors, whereby 12 studies scored 6 points, 6 studies scored 5 points, 3 studies scored 4 points, and only 1 study scored 3 points.

**Table 2:** Risk of bias summary Judgements about each risk of bias item for each included study.

**References**	**Random sequence generation (selection bias)**	**Allocation concealment (selection bias)**	**Blinding of participants and personnel (performance bias)**	**Blinding of outcome assessment (detection bias)**	**Incomplete outcome data (attrition bias)**	**Selective reporting (reporting bias)**	**Other bias**
Aaron (2019)	Low risk	Low risk	Low risk	Low risk	Low risk	Low risk	Low risk
Abutair (29)	Low risk	High risk	Low risk	Low risk	Unclear	Unclear	Low risk
Dall'Alba et al., (30)	Low risk	Unclear	Low risk	Low risk	Low risk	Low risk	Low risk
Allegretti et al. (25)	Low risk	Unclear	Low risk	Low risk	Low risk	Low risk	Unclear
Arrigo (2020)	Low risk	Low risk	Low risk	Low risk	Low risk	Low risk	Low risk
Gouni-Berthold et al. (27)	Low risk	Low risk	Low risk	Low risk	Low risk	Low risk	Low risk
Carmen (2019)	Low risk	Low risk	Low risk	Low risk	Low risk	Low risk	Low risk
Chang et al. (40)	Low risk	Low risk	Low risk	Low risk	Low risk	Low risk	Low risk
Daniel (2011)	Low risk	Unclear	Low risk	Low risk	Low risk	Low risk	Low risk
Eric (2008)	Unclear	Unclear	High risk	Low risk	Low risk	Low risk	Low risk
Fabiola (2014)	Low risk	High risk	High risk	Low risk	Low risk	Low risk	Low risk
Gregory (2012)	Low risk	Low risk	Low risk	Low risk	Low risk	Low risk	Low risk
Harold (2010)	Low risk	Low risk	Low risk	Low risk	Low risk	Low risk	Low risk
Jarrar et al. (31)	Low risk	Unclear	Low risk	Low risk	High risk	Low risk	Low risk
Johnston et al. (32)	Low risk	Unclear	Low risk	Low risk	Low risk	Low risk	Low risk
Karim (2020)	Low risk	Low risk	Low risk	Low risk	Low risk	Low risk	Unclear
Kassi (33)	Unclear	Unclear	Low risk	Low risk	Low risk	Low risk	Unclear
Katcher (34)	Low risk	Low risk	Low risk	Low risk	Low risk	Low risk	Low risk
Khaider (2013)	Low risk	Low risk	Low risk	Low risk	Low risk	Low risk	Low risk
Lankinen et al. (35)	Low risk	Low risk	Low risk	Low risk	High risk	Low risk	Low risk
Leão et al., (36)	Low risk	Unclear	Low risk	Low risk	High risk	Low risk	Low risk
Leber et al. (41)	Low risk	High risk	Low risk	Low risk	Low risk	Low risk	Low risk
Lefranc (37)	Low risk	Unclear	Low risk	Low risk	Low risk	Low risk	Unclear
Leila (2018)	Low risk	Low risk	Low risk	Low risk	Low risk	Low risk	Low risk
Linke et al. (28)	Low risk	Low risk	Low risk	Low risk	Low risk	Low risk	Low risk
Loek (2018)	Low risk	Low risk	Low risk	Low risk	Low risk	Low risk	Low risk
Louise (2019)	Low risk	Low risk	High risk	Low risk	Low risk	Low risk	Low risk
Luciana (2016)	Low risk	Unclear	Unclear	Low risk	Low risk	Low risk	Low risk
Martin (2018)	Low risk	Low risk	Low risk	Low risk	Low risk	Low risk	Low risk
Mocanu et al. (24)	Low risk	Unclear	Low risk	Low risk	Low risk	Low risk	Low risk
Pan et al. (42)	Low risk	Unclear	Low risk	Low risk	Low risk	Low risk	Low risk
Rikke (2012)	Low risk	Low risk	Low risk	Low risk	Low risk	Low risk	Low risk
Robertson et al. (38)	Low risk	Low risk	Low risk	Low risk	Low risk	Low risk	Low risk
Safavi et al. (44)	Low risk	Unclear	Low risk	Low risk	Low risk	Low risk	Low risk
Samira (2018)	Low risk	Low risk	Low risk	Low risk	Low risk	Low risk	Low risk
Schioldan et al. (39)	Unclear	Low risk	Unclear	Unclear	Low risk	Low risk	Unclear
Sony (2017)	Low risk	Unclear	Low risk	Low risk	Low risk	Low risk	Low risk
Tannaz (2014)	Low risk	Unclear	Low risk	Low risk	Low risk	Low risk	Low risk
Thoenes et al. (28)	Low risk	Low risk	Low risk	Low risk	Low risk	Low risk	Low risk
Tripolt et al. (43)	Low risk	Low risk	Low risk	Low risk	Low risk	Low risk	Low risk
Vanessa (2015)	Low risk	Low risk	Low risk	Low risk	Low risk	Low risk	Low risk
Vrieze (45)	Low risk	Low risk	Low risk	Low risk	Low risk	Low risk	Low risk

### Effect of Microbial Therapy on Blood Glucose Control

**Figure 2:**
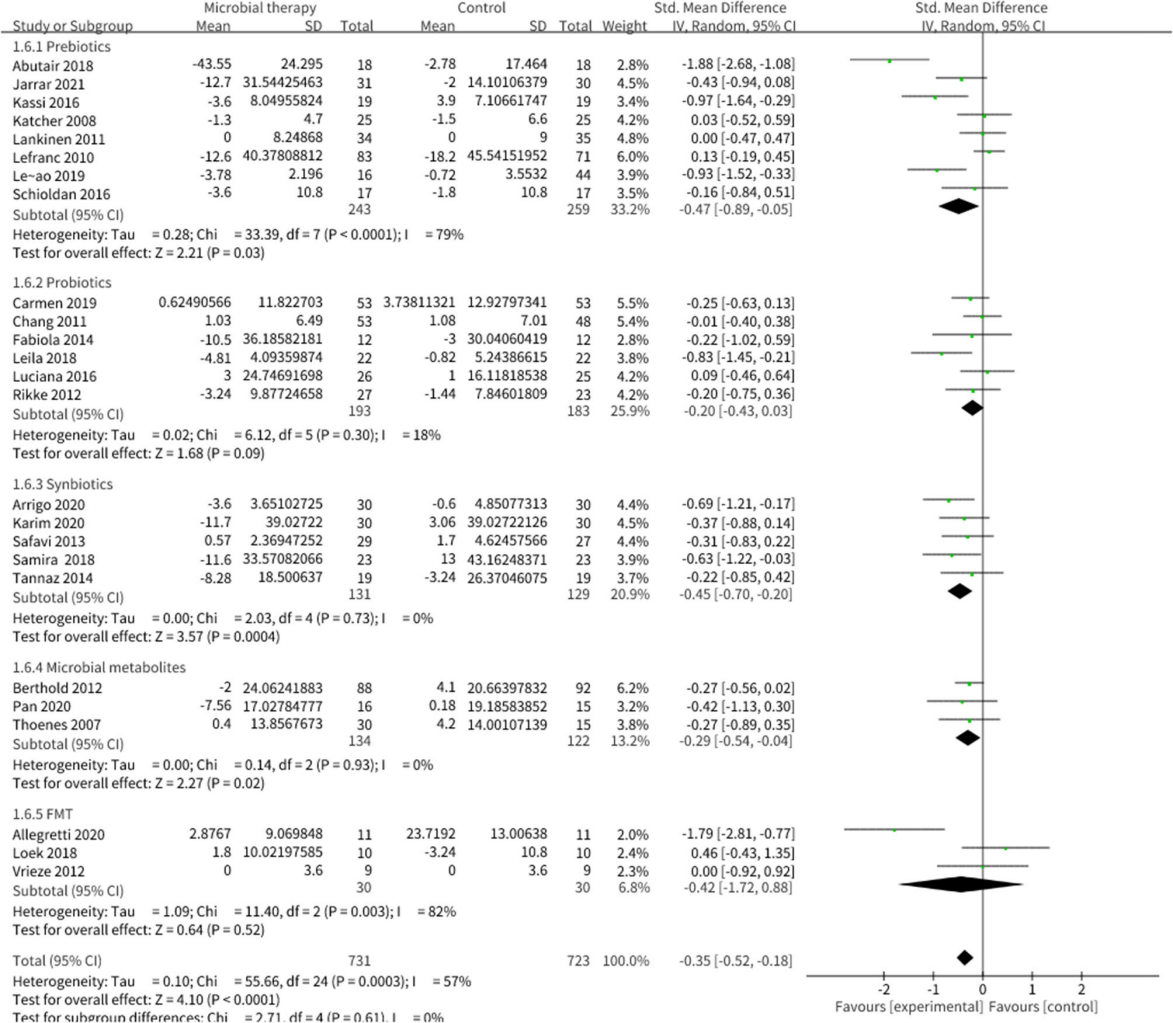
Comparison of standard mean difference (SMD) of fasting blood glucose (FBG) control between intervention groups and control groups Tau^2^ = 0.10, I^2^ = 57%, 95% CI −0.52 to −0.18, Z = 4.10, *p* < 0.0001. Significant difference was shown in FBG.

Twenty-two studies enrolling overall 1,454 participants have investigated the effect of microbial therapy on FBG (Figure 2), and an intervention group established more pronounced decline in FBG (SMD = −0.35, 95% CI −0.52, −0.18, *P* < 0.0001) with moderate heterogeneity (I^2^ = 57%). Publication bias was not reported in Begg's test (*p* = 0.141) but was reported in Egger's test (*p* = 0.026). The studies led by Abutair (29) and Allegretti et al. (25) have a high risk of bias. Even so, concomitant with the statistical decline in FBG, fasting insulin (Supplementary Figure 1) (42), which determines the ability of insulin resistance, did not show statistical difference (SMD = −0.22, 95% CI −0.49, 0.05, *P* = 0.10), similar to HOMA-IR (SMD = −0.23, 95% CI −0.49, 0.02, *P* = 0.08) (Supplementary Figure 2) (42). Sensitivity analysis indicated that when dropped one study from Luciana et al. used Bifidobacterium lactis for probiotic intervention, rather than Lactobacillus mainly in the other studies, the pooled outcome of HOMA-IR was −0.28 (95% CI −0.54, −0.03). Additionally, the result of HbA1c% (Supplementary Figure 3) (42), which reveals the level of blood glucose control in the last 3 months, was not statistically different to the control group (SMD = −0.11, 95% CI −0.50, 0.29, *P* = 0.60). This may be attributed to the short intervention duration in most studies.

### Effect of Microbial Therapy on BP Control

Eighteen studies explored the effect of microbial therapy on BP (Supplementary Figures 4, 5) (42), leading to a non-statistical difference to the placebo in SBP (SMD = −0.11, 95% CI −0.32, 0.10, *P* = 0.29) and in DBP (SMD = −0.18, 95% CI −0.39, 0.02, *P* = 0.08). Sensitivity analysis showed that removing one study led by Bernini et al. (52) could make the DBP outcome significant (SMD = −0.24, 95% CI −0.41, −0.07), whereby no study could exert excessive contribution to the SBP outcome.

### Effect of Microbial Therapy on Serum Lipoproteins Control

**Figure 3:**
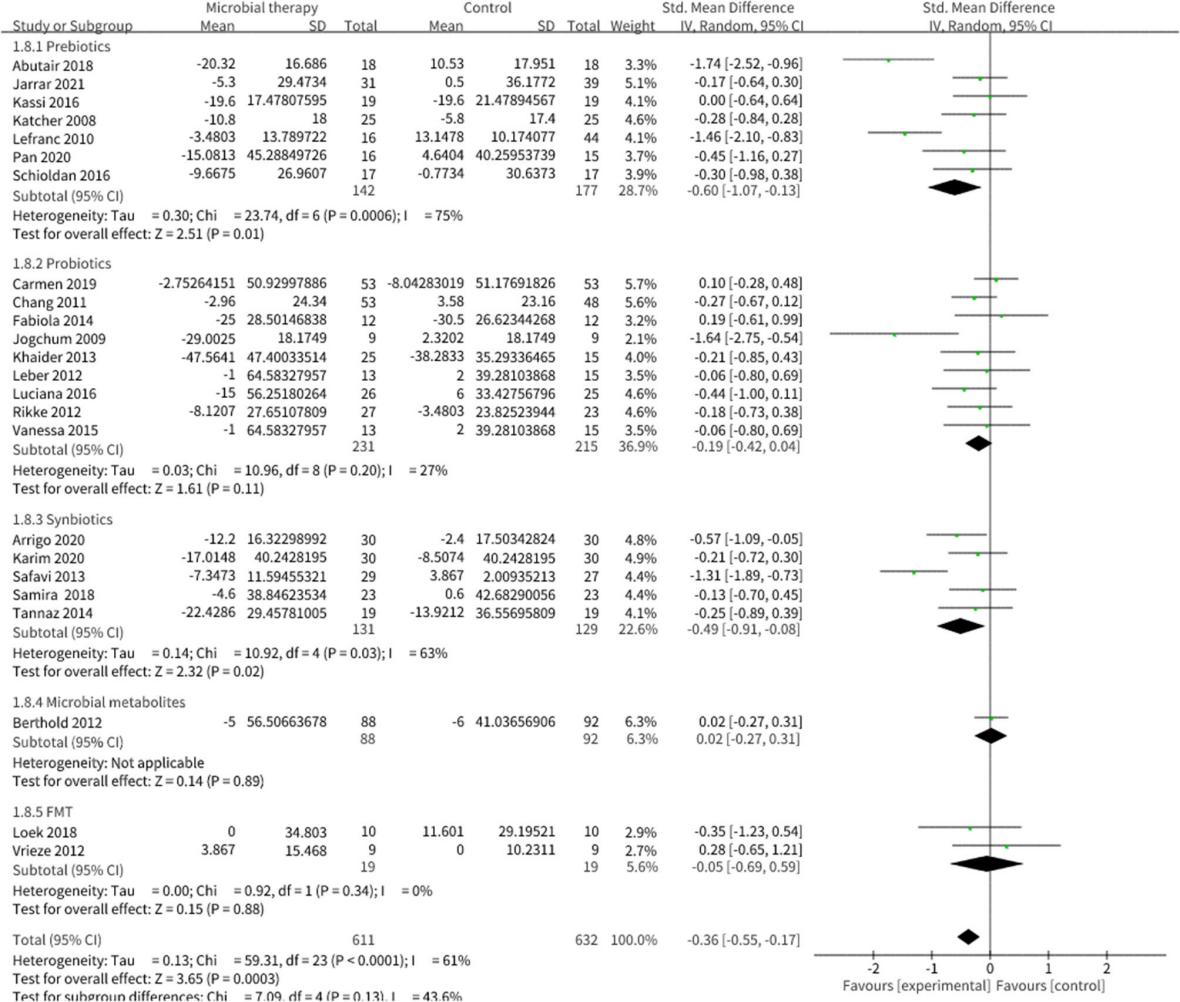
Comparison of SMD of total cholesterol (TC) control between intervention groups and control groups Tau^2^ = 0.13, I^2^ = 61%, 95% CI −0.55 to −0.17, Z = 3.65, *p* = 0.0003. Significant difference was shown in TC.

**Figure 4:**
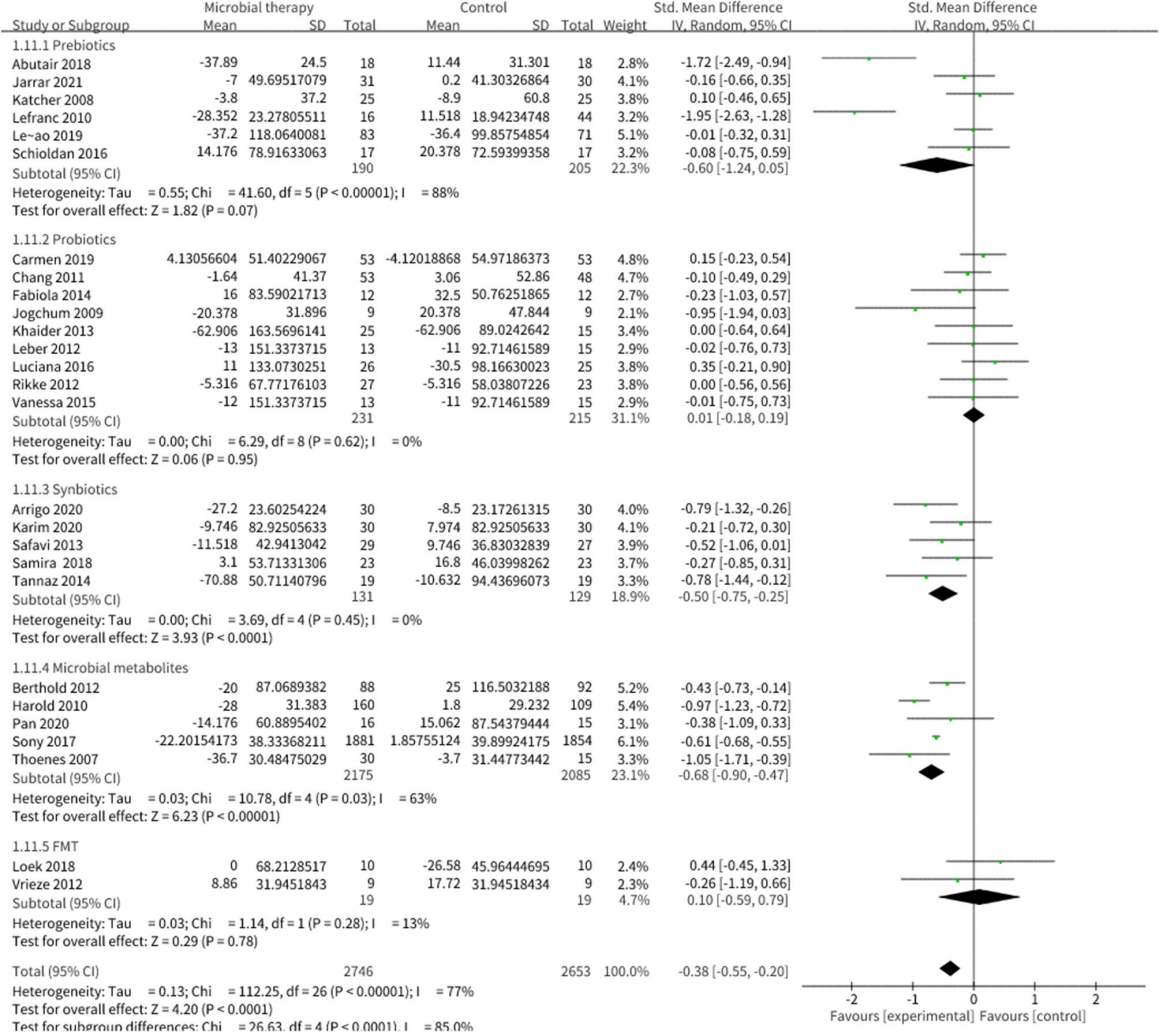
Comparison of SMD of triacylglycerol (TG) control between intervention groups and control groups Tau^2^ = 0.13, I^2^ = 77%, 95% CI −0.55 to −0.20, Z = 4.20, *p* < 0.0001. Significant difference was shown in TG.

**Figure 5:**
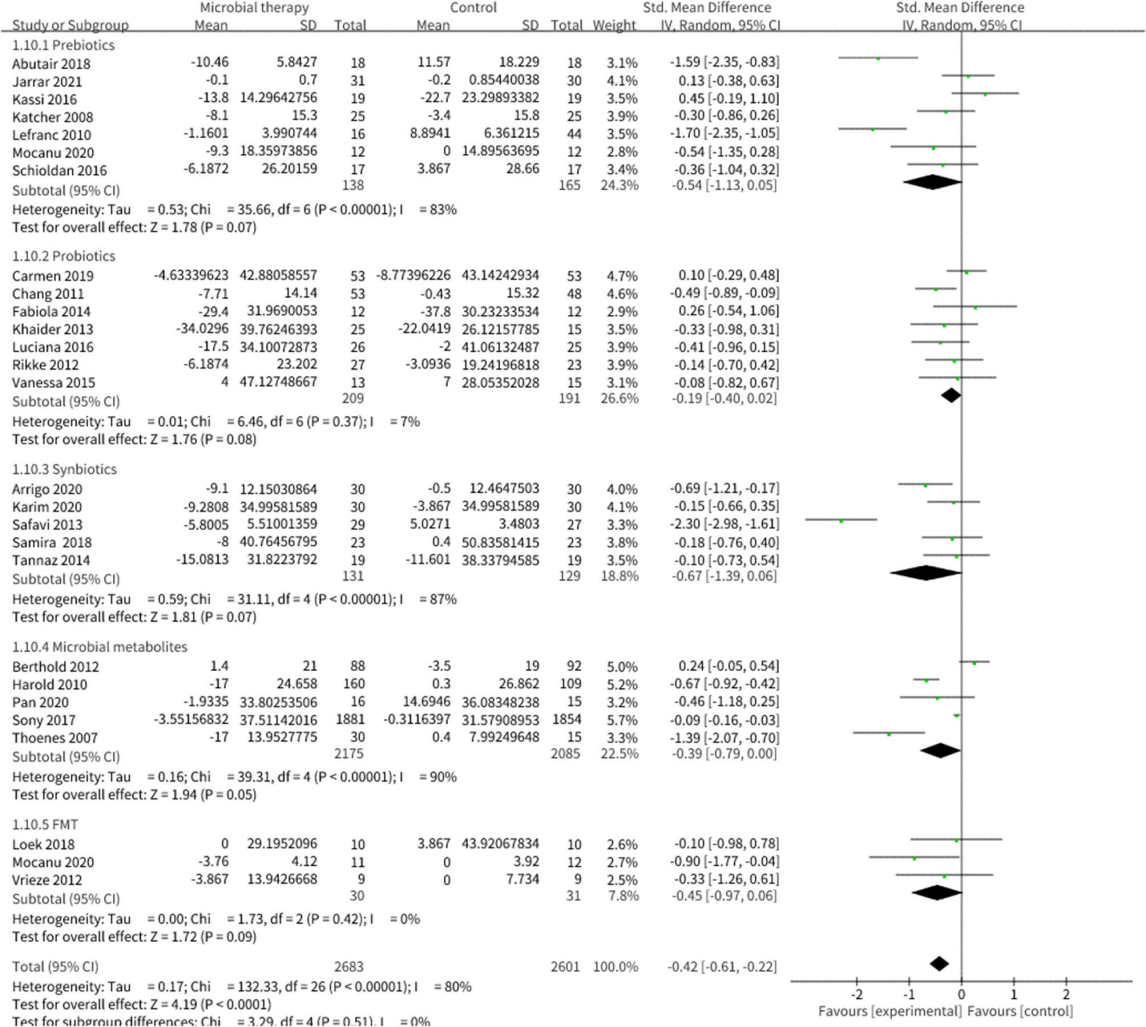
Comparison of SMD of low-density lipoprotein cholesterol (LDL-C) control between intervention groups and control groups Tau^2^ = 0.17, I^2^ = 80%, 95% CI −0.61 to −0.22, Z = 4.19, *p* < 0.0001. Significant differences were shown in LDL-C.

**Figure 6:**
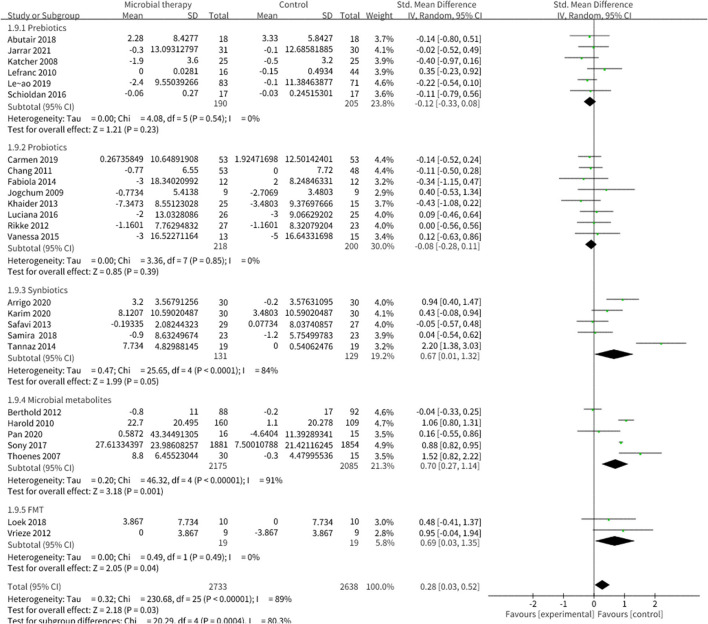
Comparison of SMD of high-density lipoprotein cholesterol (HDL-C) control between intervention groups and control groups Tau^2^ = 0.32, I^2^ = 89%, 95% CI 0.03 to 0.52, Z = 2.18, *p* = 0.03. Significant differences were shown in HDL-C.

Microbial therapy could regulate hyperlipemia to some extent, as indicated by more dampened level of TC (SMD = −0.36, 95% CI −0.55, −0.17, *P* < 0.0001) (Figure 3), TG (SMD = −0.38, 95% CI −0.55,−0.20, *P* < 0.0001) (Figure 4), LDL-C (SMD = −0.42, 95% CI −0.61, −0.22, *P* < 0.0001) (Figure 5), and more strong elevation in HDL-C (SMD = 0.28, 95% CI.03, 0.52, *P* = 03) (Figure 6) with significant heterogeneity. No publication bias was uncovered in the TC outcome by Begg's test (*p* = 0.771) and Egger's test (*p* = 0.136), similar to the TG outcome (Begg's test *p* = 0.508, Egger's test *p* = 0.069). In the HDL-C outcome, there was no hint of publication bias by Begg's test (*p* = 0.072) unlike in Egger's test (*p* = 0.001), which is similar to the LDL-C outcome (Begg's test *p* = 0.182, Egger's test *p* = 0.022).

### Effect of Microbial Therapy on Anthropometric Parameters

**Figure 7:**
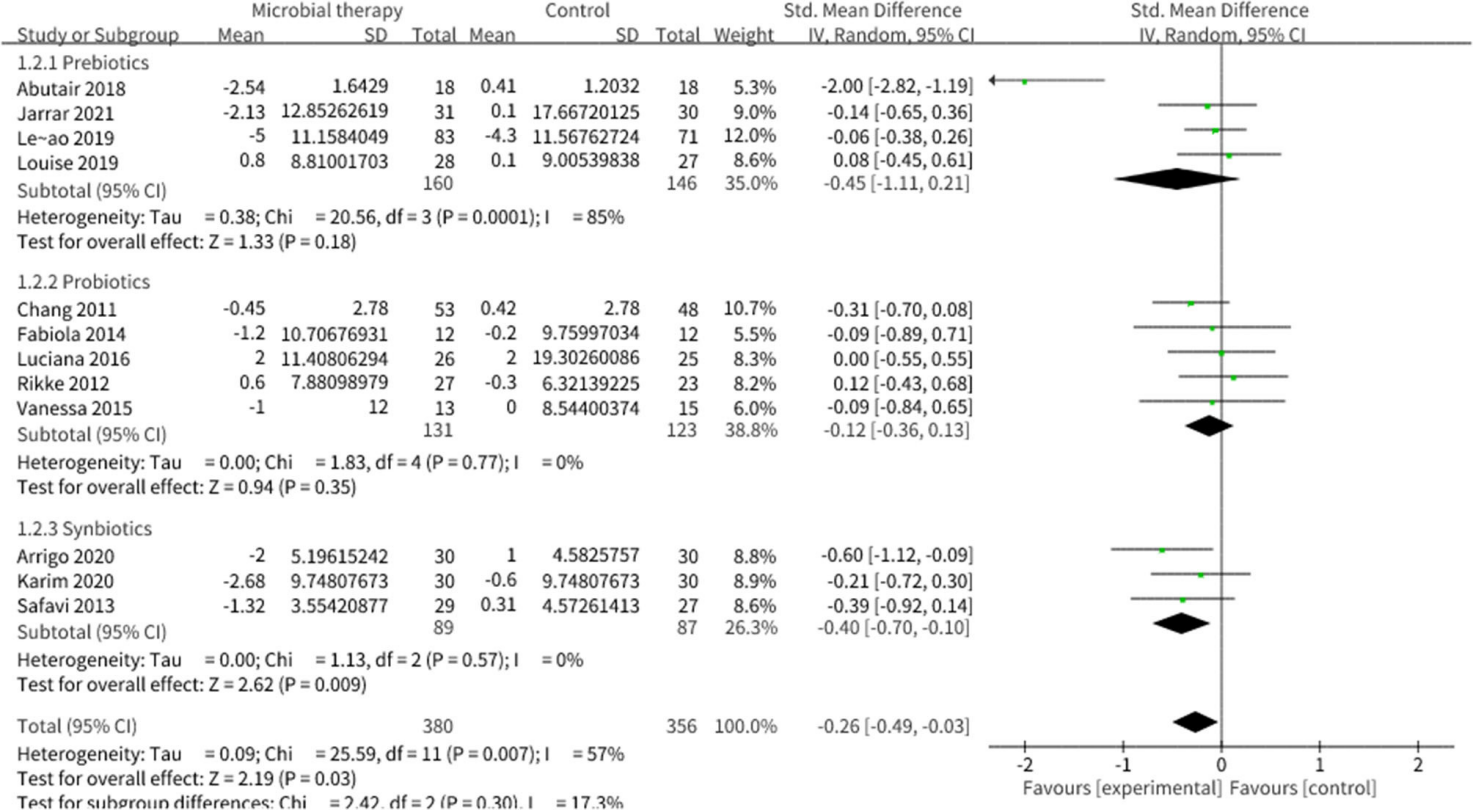
Comparison of SMD of anthropometric parameters between intervention groups and control groupsTau^2^ = 0.09, I^2^ = 57%, 95% CI −0.49 to −0.03, Z = 2.19, *p* = 0.03. Significant difference was shown in WC.

Twenty studies reported the effect of microbial therapy on WC (Figure 7). A more pronounced decline was displayed to the placebo (SMD = −0.26, 95% CI −0.49, −0.03, *P* = 0.03) with moderate heterogeneity (I^2^ = 57 %, *p* = 0.007). No publication bias was assessed by Begg's test (*p* = 0.731) and Egger's test (*p* = 0.231). No significant difference was displayed compared with the placebo in BMI (SMD = −0.13, 95% CI −0.27, 0.00, *P* = 0.05) (Supplementary Figure 6) (42). Sensitivity analysis indicated that when we removed the study conducted by Leão et al. (36), which utilized oat bran as prebiotic intervention, the pooled result BMI could be significant (SMD = −0.16, 95% CI −0.31, −0.01).

### Adverse Events

Treatment-related adverse experiences could be attributed to the nature of the interventions. In microbial metabolites, niacin-induced flushing was reported in three studies (28, 60, 61), as well as the slight gastrointestinal (27)and hepatic (60) disorders. For probiotics and prebiotics, gastrointestinal symptoms, including increased bowel movements, diarrhea, flatulence, temporary constipation, and decreased appetite were mentioned (30, 36, 41, 49). The study led by Louise et al. (36) in 2019 also reflected seasonal diseases such as sore throat, common cold, and influenza. Seven serious events with no specific indication were even recorded in Gouni-Berthold's trial (27).

## Discussion

Our analysis showed that microbial therapy is essential for mounting an effective response against intertwined metabolism in MetS. Building on the pooled outcomes, we provided strong evidence that microbial therapy application significantly dampens the risk indicators in MetS, including FBG, TC, TG, HDL-C, LDL-C, and WC. Further showing the straight benefit of microbial therapy in MetS is the improvement of DBP, HOMA-IR, and BMI in a sensitivity analysis. After omitting one study using *Bifidobacteriumlactis* as probiotic intervention, DBP and HOMA-IR improvements showed statistical significance, whereby BMI decreased significantly after neglecting one study that employed oat bran as prebiotic intervention. No obvious publication bias was detected in most of the bias test that we performed.

About 100 trillion micro-organisms inhabit the human gastrointestinal tract, providing unique metabolic functions to the host and giving fundamental importance to health and disease (68, 69). Early in 2007, animal studies demonstrated that a high-fat diet could chronically increase the proportion of lipopolysaccharide (LPS) contained in the gut together with the elevation of inflammation markers, liver triglyceride content, and liver insulin resistance (70), thereby contributing to the emergence of gut-centric theory in MetS. Evidence suggested that ingestion of a high-fat and low-fiber diet could induce the dysbiosis of gut microbiome, which contributed to the aberrant blooms or loss of bacteria (71). Of these intertwined bacteria, the proportion of gram-negative microbiota (mainly *Bacteroidetes* and *Proteobacteria*) (72) was notably elevated, while the relative proportions of gram-positive microbiome including *Lactobacillus* and *Bifidobacterium* were notably decreased. As consequence, aberrant metabolites from maladjusted bacteria, such as lipopolysaccharide (LPS) and trimethylamine (TMA), could disrupt intestinal barrier integrity, which should have been maintained by homeostatic metabolites such as glucagon-like peptide 1 (GLP-1) and GLP-2 (71). When these metabolites circulated into the liver, adipose, and other tissues, endoplasmic reticulum stress in lipid-overloaded adipocytes (73), and/or innate immune Toll-like receptors (TLRs) that signal activation (70) would be invited, leading to the chronic low—grade systematic inflammation (74). Consequently, this chronic inflammation would ultimately bring about metabolism perturbation (75), introducing the occurrence of MetS. The essential role of gut barrier integrity in chronic systematic inflammation attributes microbiome to the core in the inflammation-induced metabolic defects.

However, this ensuing chronic systematic inflammation and dysmetabolism could be mediated by microbiome modulation. Probiotics or FMT are conductive to restore disordered microbial function in alleviating obesity, blood lipids, and even inflammation in patients (50, 52). Through our systematic retrieval, we discovered that *Lactobacillus* and *Bifidobacterium* are the most commonly utilized probiotic interventions and displayed anticipated benefits. As mentioned above, patients with MetS showed a sharp decline of gram-positive bacteria but also an increase in gram-negative bacteria. Specific gram-positive bacteria, like bile salt-hydrolyzing *Lactobacillus reuteri* strain, can inhibit lipoprotein lipase, the enzyme responsible for TG hydrolysis, and, therefore, against the calorie's uptake from gut and storage in adipose tissue (72). Moreover, *Lactobacillus* also inhibit angiotensin I-converting enzyme (ACE) activities *via* casein degradation (76, 77), thus, controlling the increase of BP. Additionally, gram-positive microbiota (mainly *Lactobacillus* and *Bifidobacterium*) could degrade complex plant-derived polysaccharides (78) to SCFAs. Subjects that were assigned to be given the small intestinal infusions of allogenic microbiota have showed elevated levels of butyrate-producing intestinal microbiota, along with the increased insulin sensitivity of recipients (45).

In terms of microbial metabolism, the SCFAs of metabolites and nicotinic acid have received great attention. SCFAs serve microbial cross-feeding communities and satisfy some of our daily energy requirements (79). Moreover, they could regulate the immune system through the free fatty acid receptor FFA2R activation (80) and nuclear factor (NF)-kB inhibition (81). In addition, they suppress the lipopolysaccharide-stimulated tumor necrosis factor (TNF)α production from neutrophils (82) and the proinflammatory cytokines formation in human adipose tissue (83). Acetate, propionate, and butyrate represent the most capable SCFAs, and among them, propionate is mainly a substrate for gluconeogenesis, whereas acetate and butyrate are primarily ready for lipogenesis (84). Butyrate, as the principal fuel for intestinal epithelial cells (85), establishes a strong ability to restore gut permeability through activating peroxisomal proliferator-activated receptor (86) and upregulates mucin-associated genes (MUC1-4) expression in intestinal epithelial goblet cells (87). Eventually, the abnormally increased intestinal permeability could be alleviated. Moreover, SCFAs could influence appetite and satiety signals. The intestine expressed some proteins involved in food intake, including peptide YY (PYY), GLP-1, glucose-dependent insulinotropic polypeptide (GIP), the expression of which were induced by SCFAs and mediated by G protein-coupled receptors (Gpr) 43 and Gpr41 (72). SCFAs supplementation could foster the homeostasis of these peptides, sequentially increasing satiety levels and ultimately reducing food and energy intake (88). In line with the results, recent work demonstrated that colonic infusions of SCFAs mixtures in concentrations and ratios reached after fiber intake can increase fat oxidation, energy expenditure, and PYY, and can decrease lipolysis in overweight/obese men (89).

It is worth noting that in our analysis, most studies, including performance evaluation of microbial metabolites, used niacin as an intervention. Niacin supplementation was sufficient to significantly modulate FBG, TG, and HDL-C. According to existing work, niacin could decrease free fatty acids (FFA) concentrations in humans (65); the raise of which could cause a release of inflammatory cytokines and impairment in brachial artery flow-mediated dilation (90). This process targeted the nicotinamide adenine dinucleotide axis *via* stimulation of the salvage pathway and also supported a microenvironment for beneficial expansion of adipocytes and activation state of the resident and recruited macrophages in white adipose tissue. Therefore, this is against the low-grade inflammatory state in the high-fat-diet-induced MetS as introduced by dysfunctional white adipose tissue (91–93).

Prebiotics are non-viable food components that can be fermented by commensal organisms. They could be converted into SCFAs and other beneficial microbial metabolites through bacteria fermentation. Supplementation of prebiotics could create an acidic milieu in the gut, suppressing the growth of pathogenic or opportunistic pathogenic bacteria such as *Clostridium perfringens* and *Escherichia coli* (94), however, preferentially stimulating the growth of specific bacteria strains like *Lactobacillus* and *Bifidobacterium* (95, 96). Different prebiotics exhibit variant metabolism-regulating effects. From existing pieces of research, the relative solubility of different oligosaccharides or polysaccharides related with cell wall material shared variable digestion rate by bacteria desorbed from the biofilms, and followed by the discrepant SCFAs generation (85). SCFAs serve as initial substrates for hepatic gluconeogenesis and *de novo* lipogenesis (72), thereby affecting the metabolic results. Therefore, we attributed it to be responsible for our sensitivity analysis result; our study utilized oat bran as a prebiotic intervention, thus setting this as the main factor that influenced the significance of the pooled BMI result.

Our observation that the pooled HOMA-IR and DBP became significant after eliminating the study led by Luciana using *Bifidobacteriumlactis* as probiotic intervention seems to be attributed to the differential ability in carbohydrate metabolism (97). The characteristic types of glycosyl hydrolases in these two bacteria reflected the different types of oligosaccharides that can be fermented (*Lactobacillus* and *Bifidobacterium* digest plant and animal-oriented sugars, respectively) (97). In addition, a high diversity impacting on glucose control by specific species of microbiome from *Lactobacillus* (98, 99) and *Bifidobacterium* (100, 101) was also reported. Admittedly, some of the variances were accounted for by a different approach of an outcome data presentation in Luciana's article, which was manifested as median (25–75%), while the other works mainly utilized mean (SD) or mean (SE).

There are other similar integration studies focused on this subject. In 2016, Sáez-Lara reviewed the effects of probiotics and synbiotics on metabolism-related diseases including MetS, and have reported decreased plasma lipid levels (102). In the same year, Chen et al. (103) suggested an inverse association between dietary fiber intake and the risk of MetS. However, Dong et al. (104) denoted that probiotic treatment alone could not reduce overall health risks in MetS. A similar conclusion was recapitulated with the study by Snelsonet al. (105) through resistant starch intervention. Collectively, existing articles mostly studied the specific species of microbial therapy on MetS with inconsistent conclusions. Hence, this analysis summarized the relevant treatment of MetS and outlined the importance of microbial therapy to improve risk factors for patients affected by MetS. Therefore, this study provided further evidence to the causes of MetS and the core role of microbiome in systematic diseases.

## Conclusion and Limitations

From this analysis, conditioning with microbial therapy presented a favorable effect in controlling BG, blood lipid, and BP. The effect of attenuation in dysmetabolism may be beneficial in the long term for the improvement of MetS or other metabolism-related diseases like diabetes and even other diseases. Due to the relatively single microbial metabolites intervention and the existence of variables like experimental design, the data should be extrapolated more prudently, and further RCTs in various microbial therapy are urgently needed before clinical application.

## Data Availability Statement

The original contributions presented in the study are included in the article/Supplementary Materials, further inquiries can be directed to the corresponding author/s.

## Author Contributions

XL and ZX: study design and literature search strategy. FW and JS: initial manuscript screening. JS and YC: data extraction and verification. DS and GR: risk-of-bias assessment. BP: statistical analysis. BP and XL: writing of first draft of the manuscript YH and CX: manuscript revision. All authors contributed to the article and approved the submitted version.

## Funding

This work was supported by grants from the Zhejiang Provincial Natural Science Foundation of China [LY20H180010], the Wenzhou Science and Technology Bureau [Y20180142], the Wenzhou Science and Technology Bureau [Y2020214], the Zhejiang Provincial Medical and Health Science and Technology Project General Project [No. 2019KY461], the Wenzhou Science and Technology Bureau [Y20190060], and the Zhejiang Provincial Public Welfare Technology Research Plan/Social Development Project [LGF20H070003].

## Conflict of Interest

The authors declare that the research was conducted in the absence of any commercial or financial relationships that could be construed as a potential conflict of interest.

## Publisher's Note

All claims expressed in this article are solely those of the authors and do not necessarily represent those of their affiliated organizations, or those of the publisher, the editors and the reviewers. Any product that may be evaluated in this article, or claim that may be made by its manufacturer, is not guaranteed or endorsed by the publisher.

## Abbreviations

MetS: metabolic syndrome
RCTs: randomized controlled trials
WC: waist circumference
SMD: standard mean difference
FBG: fasting blood glucose
TC: total cholesterol
LDL—C: low—density lipoprotein cholesterol
TG: triacylglycerol
HDL—C: high—density lipoprotein cholesterol
DBP: diastolic blood pressure
HOMA—IR: Homeostatic Model Assessment of Insulin Resistance
BMI: body mass index
SBP: systolic blood pressure
HbA1c%: hemoglobin A1c
FMT: fecal microbiota transplantation
SCFAs: short-chain fatty acids.
