# Supplementary material for: A Meta-Analysis of Microbial Therapy Against Metabolic Syndrome: Evidence From Randomized Controlled Trials
Source: Front Nutr. 2021 Dec 15;8:775216. doi: 10.3389/fnut.2021.775216 (PMC8714845; doi:10.3389/fnut.2021.775216)
Supplement: Supplementary file 1 [file Data_Sheet_1.docx]

Supplementary Material

**Abbreviations**

MetS, metabolic syndrome; RCTs, randomized controlled trials; WC, waist circumference; SMD, standard mean difference; FBG, fasting blood glucose; TC, total cholesterol; LDL - C, low - density lipoprotein cholesterol; TG, triacylglycerol; HDL - C, high - density lipoprotein cholesterol; DBP, diastolic blood pressure; HOMA - IR, Homeostatic Model Assessment of Insulin Resistance; BMI, body mass index; SBP, systolic blood pressure; HbA1c%, hemoglobin A1c; FMT, fecal microbiota transplantation; SCFAs, short - chain fatty acids.

**Supplementary Figure 1:** Comparison of SMD of fasting insulin control between intervention groups and control groups

**Supplementary Figure 2:** Comparison of SMD of HOMA-IR control between intervention groups and control groups

**Supplementary Figure 3:** Comparison of SMD of HbA1c% control between intervention groups and control groups

**Supplementary Figure 4:** Comparison of SMD of SBP control between intervention groups and control groups

**Supplementary Figure 5:** Comparison of SMD of DBP control between intervention groups and control groups

**Supplementary Figure 6:** Comparison of SMD of BMI control between intervention groups and control groups

**Figure legends：**

**Supplementary Figure 1: Comparison of SMD of fasting insulin control between intervention groups and control groups** Fasting insulin: Tau^2^ = 0.16, I^2^ = 66%, 95% CI –0.49 to 0.05, Z = 1.62, p = 0.10. No significant difference was shown in fasting insulin.

**
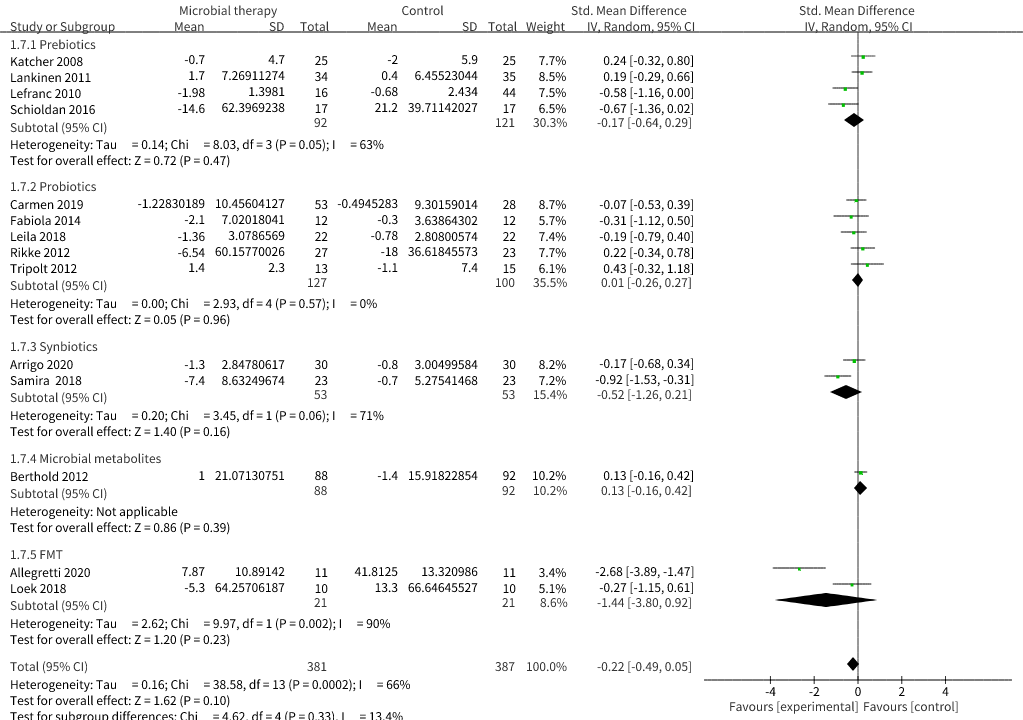
**

**Supplementary Figure 2: Comparison of SMD of HOMA-IR control between intervention groups and control groups** Tau^2^ = 0.17, I^2^ = 68%, 95% CI –0.49 to 0.02, Z = 0.1.78, p = 0.08. No significant difference was shown in HOMA-IR.


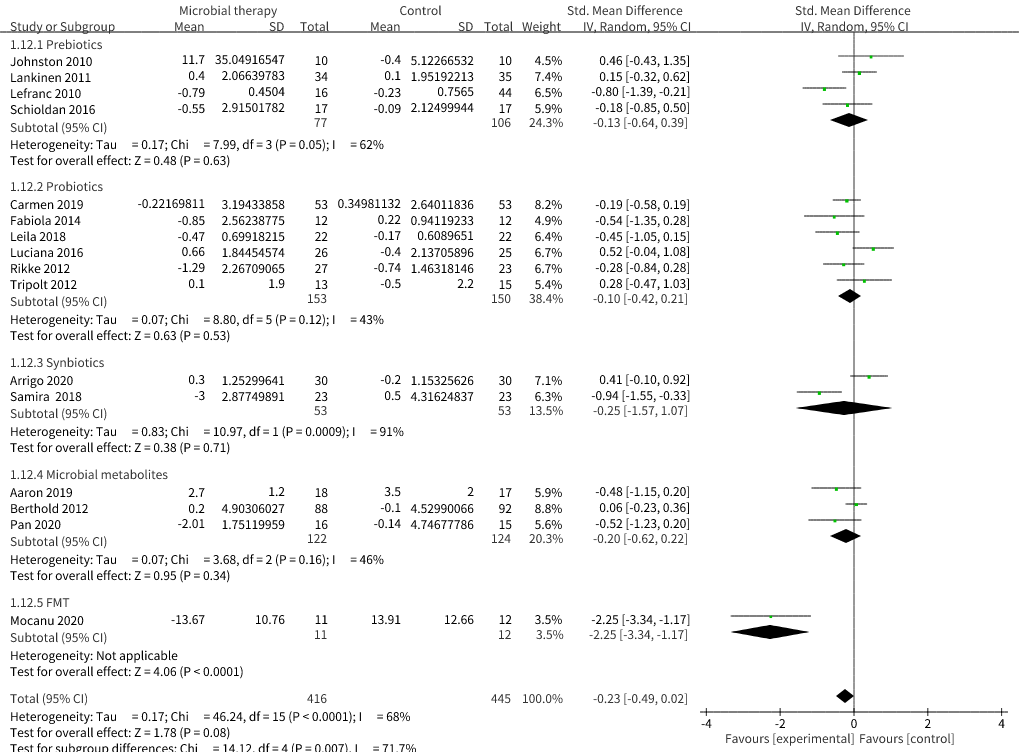


**Supplementary Figure 3:** **Comparison of SMD of HbA1c% control between intervention groups and control groups** Tau^2^ = 0.16, I^2^ = 70%, 95% CI –0.50 to 0.29, Z = 0.52, p = 0.60. No significant difference was shown in HbA1c%.


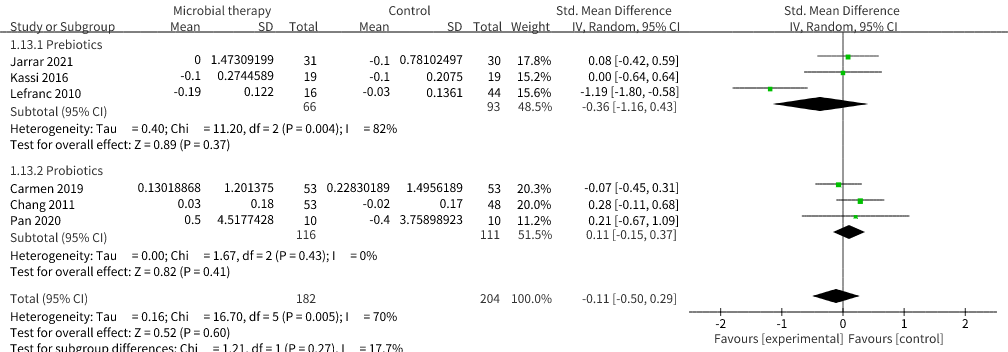


**Supplementary Figure 4: Comparison of SMD of SBP control between intervention groups and control groups** Tau^2^ = 0.13, I^2^ = 64%, 95% CI –0.32 to 0.10, Z = 1.05, p = 0.29. No significant difference was shown in SBP.


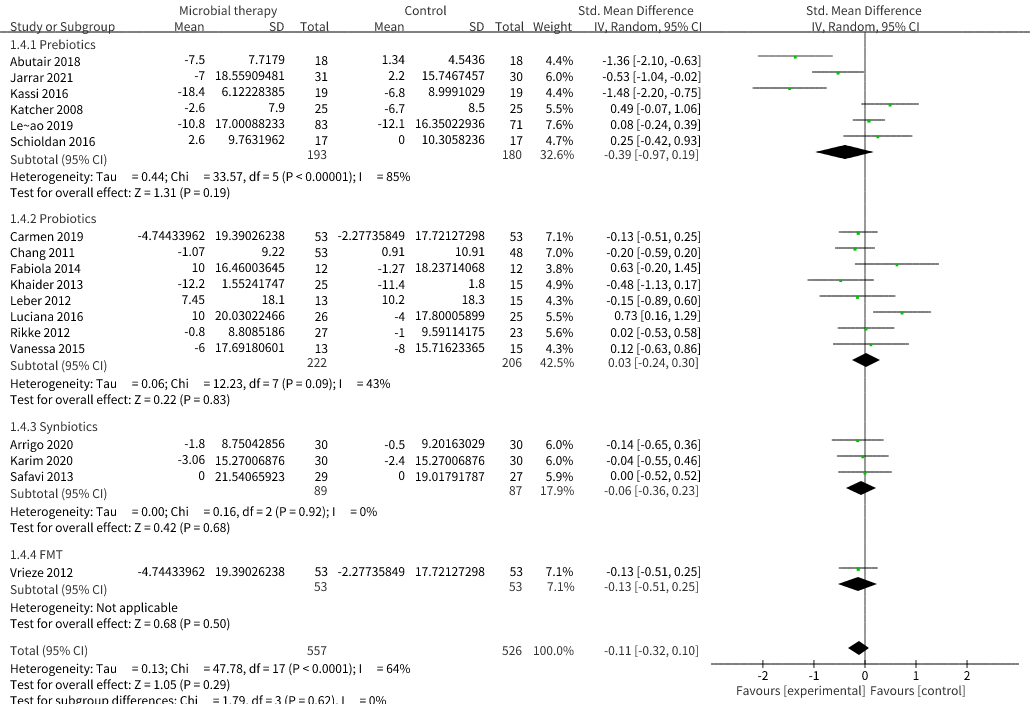


**Supplementary Figure 5: Comparison of SMD of DBP control between intervention groups and control groups** Tau^2^ = 0.12, I^2^ = 60%, 95% CI –0.39 to 0.02, Z = 1.77, p = 0.08. No significant difference was shown in DBP.


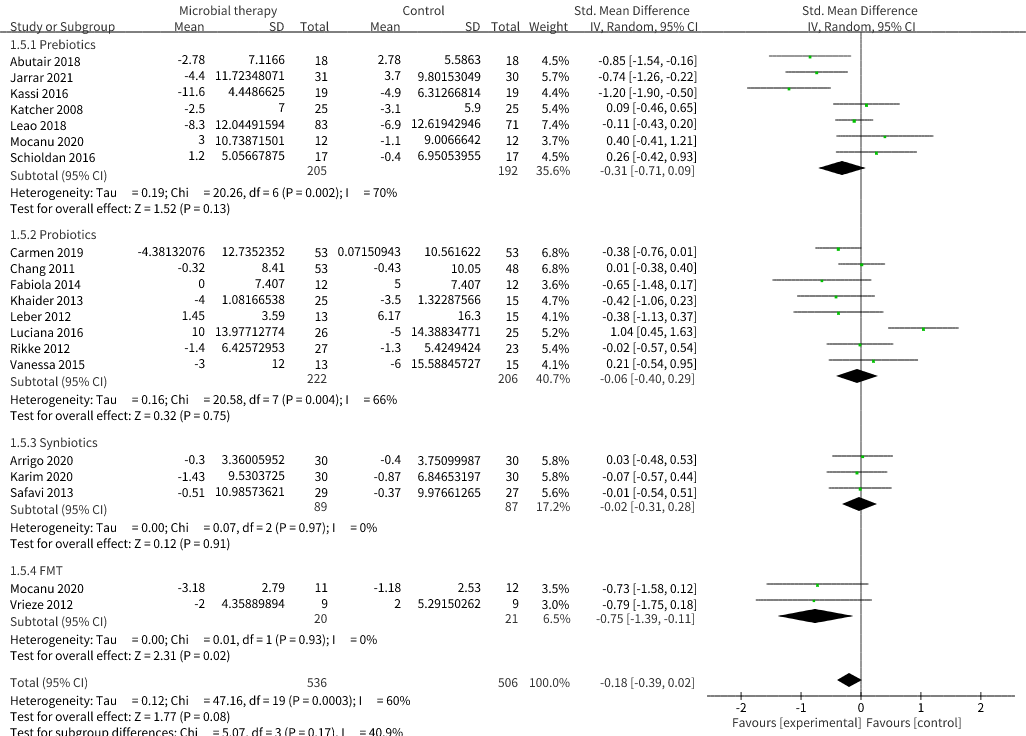


**Supplementary Figure 6: Comparison of SMD of BMI control between intervention groups and control groups** Tau^2^ = 0.00, I^2^ = 0%, 95% CI –0.27 to 0.00, Z = 1.94, p = 0.05. No significant difference was shown in BMI.

**
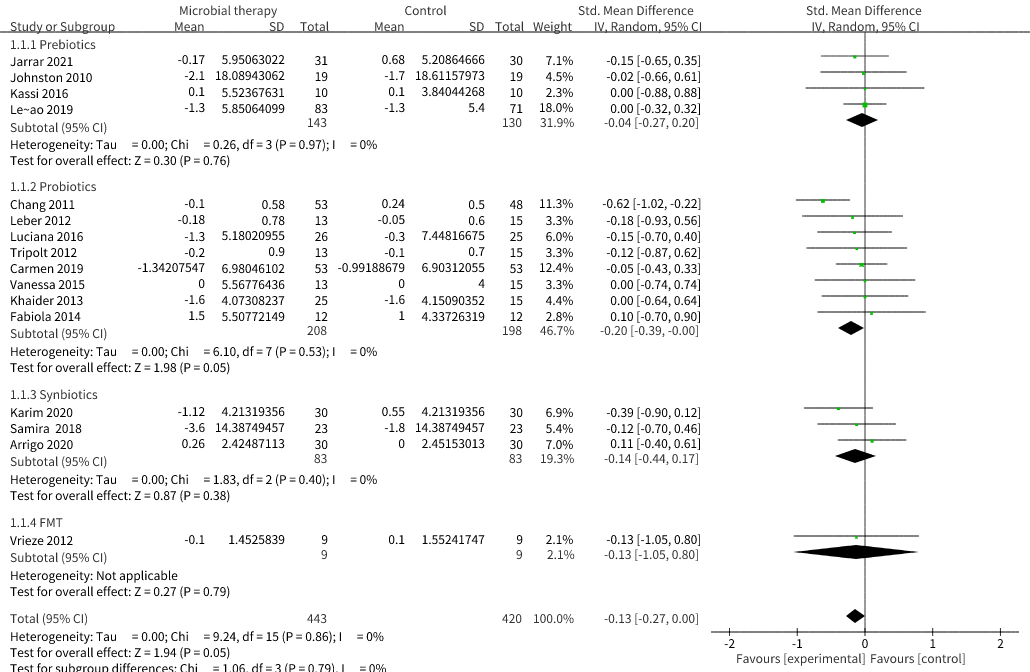
**
